# A Novel Pathway that Links Caveolin-1 Down-Regulation to BRCA1 Dysfunction in Serous Epithelial Ovarian Cancer Cells

**DOI:** 10.18650/2376-046x.11004

**Published:** 2014

**Authors:** Jingyao Xu, Stephanie Agyemang, Yunlong Qin, Kartik Aysola, Mercedes Giles, Gabriela Oprea, Ruth M O’Regan, Edward E Partridge, Sandra Harris-Hooker, Valarie Montgomery Rice, E. Shyam P Reddy, Veena N Rao

**Affiliations:** 1 Cancer Biology Program, Department of OB/GYN, Georgia Cancer Center for Excellence, Grady Health System, Atlanta, GA 30303, USA; 2 Department of Pathology and Medicine, Morehouse School of Medicine, 720 Westview Drive, SW, Atlanta, GA 30310, USA; 3 Emory University School of Medicine, Atlanta, GA, USA; 4 Department of Obstetrics & Gynecology, Division of Gynecological Oncology, 618 20th Street South, University of Alabama at Birmingham, USA

**Keywords:** BRCA1, BRCA1a, Ubc9, Serous Epithelial Ovarian Cancer, Caveolin-1, Protein-protein

## Abstract

Ovarian cancer is the second most common gynecological cancer and the five-year survival rate is only about 40%. High-grade serous carcinoma is the pre-dominant histotype associated with hereditary ovarian cancer and women with inherited mutations in BRCA1 have a lifetime risk of 40–60%. BRCA1 and its isoform BRCA1a are multifunctional proteins that are the most evolutionary conserved of all the other splice variants. Our group has previously reported that BRCA1/1a proteins, unlike K109R and C61G mutants, suppress growth of ovarian cancer cells by tethering Ubc9. In this study we found wild type BRCA1/1a proteins to induce expression of caveolin-1, a tumor suppressor in BRCA1-mutant serous epithelial ovarian cancer (SEOC) cells by immunofluorescence analysis. The K109R and C61G disease associated mutant BRCA1 proteins that do not bind Ubc9 were not as efficient in up-regulation of caveolin-1 expression in SEOC cells. Additionally, immunofluorescence analysis showed BRCA1/1a proteins to induce redistribution of Caveolin-1 from cytoplasm and nucleus to the cell membrane. This is the first study demonstrating the physiological link between loss of Ubc9 binding, loss of growth suppression and loss of Caveolin-1 induction of disease-associated mutant BRCA1 proteins in SEOC cells. Decreased Caveolin-1 expression combined with elevated Ubc9 expression can in the future be used as an early biomarker for BRCA1 mutant SEOC.

## Introduction

Ovarian cancer is the second most common gynecological cancer and over 95 percent of malignant tumors are of the epithelial type. Epithelial cancer of the ovary is a malignant transformation of the epithelium of the surface of the ovary, peritoneum, or uterine tube [1]. Approximately 10% of the epithelial ovarian cancers (EOC) are caused by mutations in the tumor suppressor gene BRCA1 [2,3]. In sporadic EOC, BRCA1 mutations are rare, but reduced expression or aberrant subcellular localization of BRCA1 is common [4–6]. The majority of epithelial ovarian cancers (EOC) are of the serous subtype and they are further subdivided into high-grade and low-grade tumors. High-grade serous carcinoma is the predominant histotype associated with hereditary ovarian cancer and women with inherited mutations of BRCA1 have a lifetime risk of 40–60 % [7] African-American women are less likely to receive recommended surgery and chemotherapy for advanced epithelial ovarian cancer. Incomplete treatment is correlated with decreased survival and, between 1975 and 2005, the 5-year survival rate for United States white women with advanced ovarian cancer improved from 37% to 45% but declined for black women from 43% to 38% [8].

Our lab has identified and cloned two major isoforms of BRCA1, namely BRCA1a/p110 and BRCA1b/p100 [9,10], which are the most evolutionary conserved of all the isoforms and expressed at reduced levels in ovarian cancers compared to normal cells [11–14]. We found BRCA1a protein to induce apoptosis and inhibit in vivo tumor growth of hormone-independent ES-2 ovarian cancer cells, but the mechanism of tumor suppression is not known [15,16]. BRCA1 and its splice variants are nuclear proteins that have several functional domains, an N-terminal RING finger domain that interacts with several proteins and two BRCA1 C-terminal domains. We have found BRCA1, BRCA1a and BRCA1b proteins to be localized in the mitochondria, and their nuclear-cytoplasmic shuttling to be a regulated process [9,14,17]. BRCA1 nuclear import and export is mediated by the action of nuclear localization signal (NLS) and nuclear export signals (NES) located in the RING domain that mediates nuclear export via association with BARD1 [18]. The BRCA1 delta 11 isoform, which lacks NLS, also enters the nucleus via the RING-domain mediated BARD1 import pathway [19]. The RING domain of BRCA1, in complex with BARD1, mediates an E3 Ubiquitin ligase activity on ER-α in-vitro [20,21]. Recent studies using an Ubiquitin ligase-deficient BRCA1 I26A mutant suggested that the Ubiquitin ligase activity is dispensable for both genomic stability as well as homology-directed repair of double-strand DNA breaks, but is required for inhibition of ER-α activity [22,23]. Post-translational modification of proteins is reversible and normal cells use this mechanism to regulate cellular proliferation [24]. SUMO (Small Ubiquitin-like modifier) modification of proteins affects several functions like stability, localization, protein-protein interactions and transcriptional regulation (reviewed by [25–27]). The SUMO modification pathway was shown to be involved in BRCA1 response to DNA damage and transcriptional repression [28,29]. We have shown that the amino-terminal domain of BRCA1, BRCA1a and BRCA1b proteins bind to SUMO-E2-conjugating enzyme Ubc9 and regulate ER-α activity by promoting its degradation in vivo [30]. This work suggested a relationship between the SUMO and Ubiquitin pathways, similar to the Ubiquitin ligase RNF4, by highlighting the biochemical function of BRCA1 as a putative SUMO-1 and Ubc9-dependent E3 Ubiquitin ligase for ER-α SUMO conjugates [31,32].

Ubc9 binding site mutations, as well as disease associated mutation in the BRCA1 RING domain (C61G), disrupted the ability to regulate Ubc9-mediated estrogen-induced ER-α transcriptional activity in breast cancer cells [30] but did not disrupt SUMO-1 binding [28] nor auto ubiquitination activity of BRCA1 [30]. Both BRCA1/BRCA1a K109R and disease associated C61G mutants, which are localized mainly in the cytoplasm, fail to inhibit the growth of breast and ovarian cancer cells [33,34]. Ubc9 has been shown to play an important role in both cancer progression and resistance to chemotherapy [35– 38]. In fact, Ubc9 was found to act as both a positive and negative regulator of proliferation and transformation of HMGA1 proteins [37].

Caveolae are invaginations in the plasma membrane 60–80nm in diameter associated with various caveolin proteins for endocytosis, signal transduction, and vesicular transport. Caveolin-1 and 2 are most highly expressed throughout the body in endothelial cells, adipocytes, smooth muscle cells, and fibroblasts, while caveolin-3 appears to be the only form found in skeletal and cardiac muscle [41,42]. Caveolin-1 can directly regulate the activity of signaling molecules within caveolae. Interaction with caveolin-1 leads to the inhibition of the basal activity of signaling molecules and their downstream pathways. When stimulated, inhibition of these molecules facilitated by caveolin-1 is halted, allowing signal propagation. Many of the proteins that interact with, transcriptionally repress, or are inhibited by caveolin-1 fall under the pro-proliferative, oncogenic, and anti-apoptotic category of molecules. These molecules include G-protein coupled receptors, protein kinase C, and receptor tyrosine kinase. Studies using caveolin-1- null cells have demonstrated that caveolin-1 inhibits cell proliferation and cell-cycle progression. Caveolin-1-null mouse embryo fibroblasts display increased proliferation rates and cell cycle progression [43]. Caveolin-1 also has genetic characteristics that may contribute to its ability to affect proliferation rates. The human caveolin-1 locus revealed that it maps to 7q31.1, adjacent to the marker D7S522, a fragile site with deletions in many tumors in cancers such as breast, prostate, and ovarian [44]. Hence, caveolin-1 is thought of as a putative tumor suppressor of which its decreased expression allows for cancer progression. Down-regulated expression of caveolin-1 is seen in metastatic breast cancer cells. Using a model of spontaneous breast metastasis, caveolin-1 appeared to be expressed in low and non-metastatic primary tumors and to be expressed at much lower levels in highly metastatic tumors [45]. Thus, in metastatic tumors, the role of caveolin-1 as a tumor suppressor is absent, allowing the tumor to spread. In a study of caveolin-1 in ovarian cancer, immunohistochemical analysis of caveolin-1 shows normal expression of caveolin-1 in the surface epithelium and in the underlying stroma of normal ovary. A similar staining is apparent in the epithelial lining of a serous tumor, although loss of the membrane-associated localization and down-regulation can be observed in a grade 1 serous carcinoma. A complete loss of caveolin-1 expression is seen in high-grade serous epithelial carcinoma [46]. BRCA1 has been shown to induce the transcriptional activation of the caveolin-1 gene in mouse embryo fibroblast cells [47]. Here, we have further investigated these findings and have studied the effect of wild type BRCA1, BRCA1a and altered Ubc9 binding BRCA1a mutants on the expression of caveolin-1 in a physiologically relevant BRCA1 mutant SEOC cells.

## Materials and Methods

### Expression Constructs

Full length BRCA1a, BRCA1a Mut #1 and BRCA1a Mut #4, were cloned into pCDNA3 vectors as described previously [30]. Point mutations were generated as described previously [30].

### Cell Culture

UWB1.289 and UWB1.289 BRCA1 cells were obtained from American Type Culture Collection (Rockville, MD, USA) and cultivated as described previously [48].

### Antibodies and Reagents

The antibodies used in this study were MS110 ascites (Ab1, EMD Millipore), polyclonal rabbit anti-caveolin-1 antibody (Santa Cruz Biotechnology).

### Immunoflourescence Analysis

To analyze the subcellular localization UWB1.289 and UWB1.289+BRCA1 cells were seeded into 6-well plates a day before transfection with pcDNA3 BRCA1a and their respective mutant plasmids. Cells were fixed in methanol 24 hrs after transfection and blocked with 10% BSA, followed by primary Monoclonal Mouse anti-caveolin-1 antibody 1:150 dilute for 1hr and Alexa488 goat anti-mouse (Molecular Probes) for 50 min, in combination with staining with DAPI dye or Hoechst. The cells were visualized under a fluorescent microscope (Olympus, 100× oil lens) as described previously [34] or LSM 700 Confocal Microscope (Carl Zeiss, 63× oil lens).

## Results

### BRCA1 and Caveolin-1 Expression is Reduced Significantly in BRCA1 Mutant SEOC Cells

**Figure 1:**
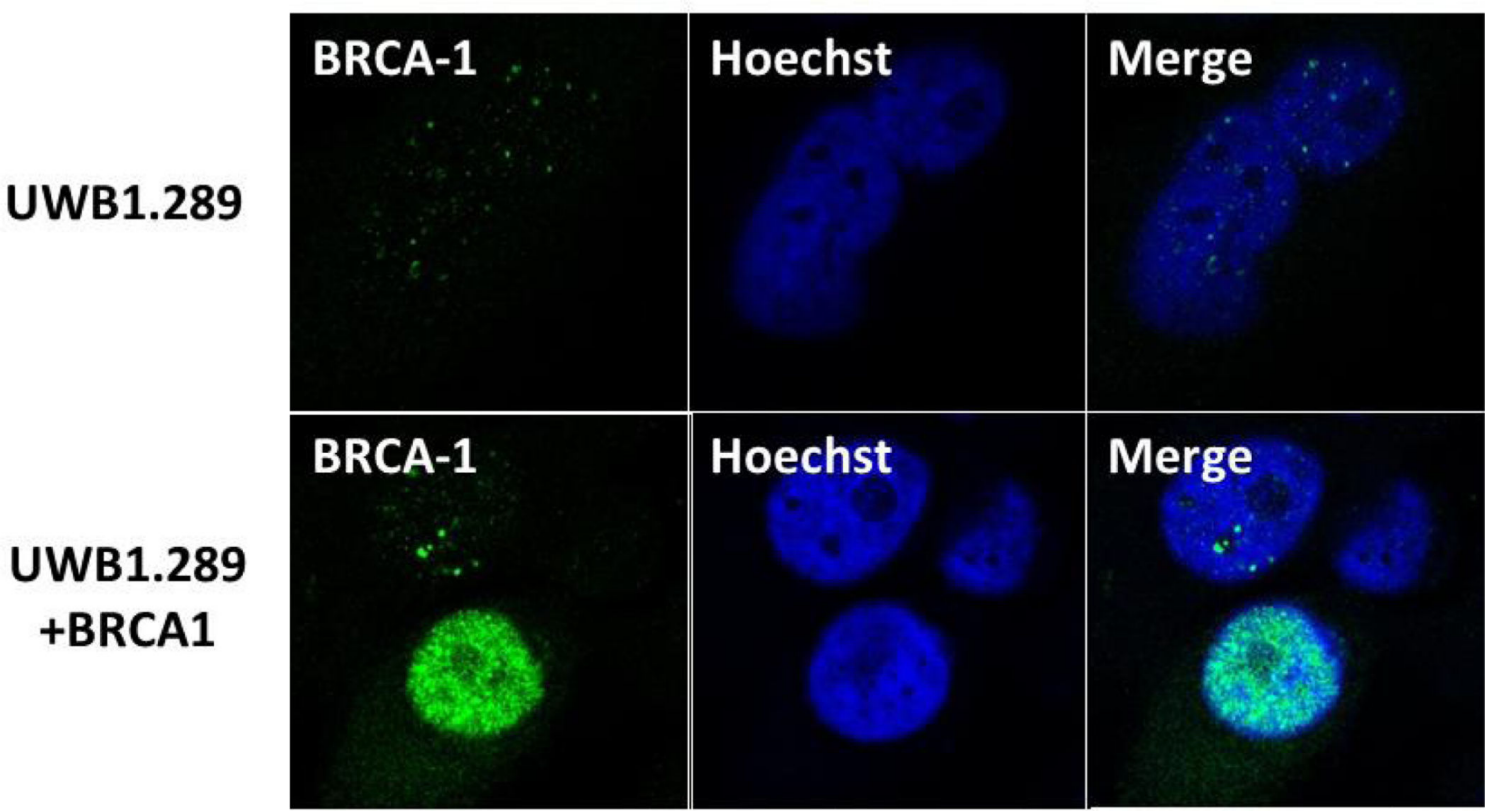
Loss of BRCA1 expression in BRCA1 mutant SEOC cell line UWB1.289 cells by immunoflourescence analysis. UWB1.289 and UWB1.289+BRCA1 cells were seeded into six-well plates and after 24 hours the nuclei were visualized with DNA staining dye Hoechst. Cells were fixed in icy methanol and probed with BRCA1 antibody (EMD Millipore, Ab1 1/100 followed by Alexa Fluor 488 labeled secondary antibody (Invitrogen, 1/200) as described previously [34]. The nuclei were visualized by Hoechst staining. The images were taken using LSM 700 Confocal Microscope 63× oil lense, Carl Zeiss).

**Figure 2:**
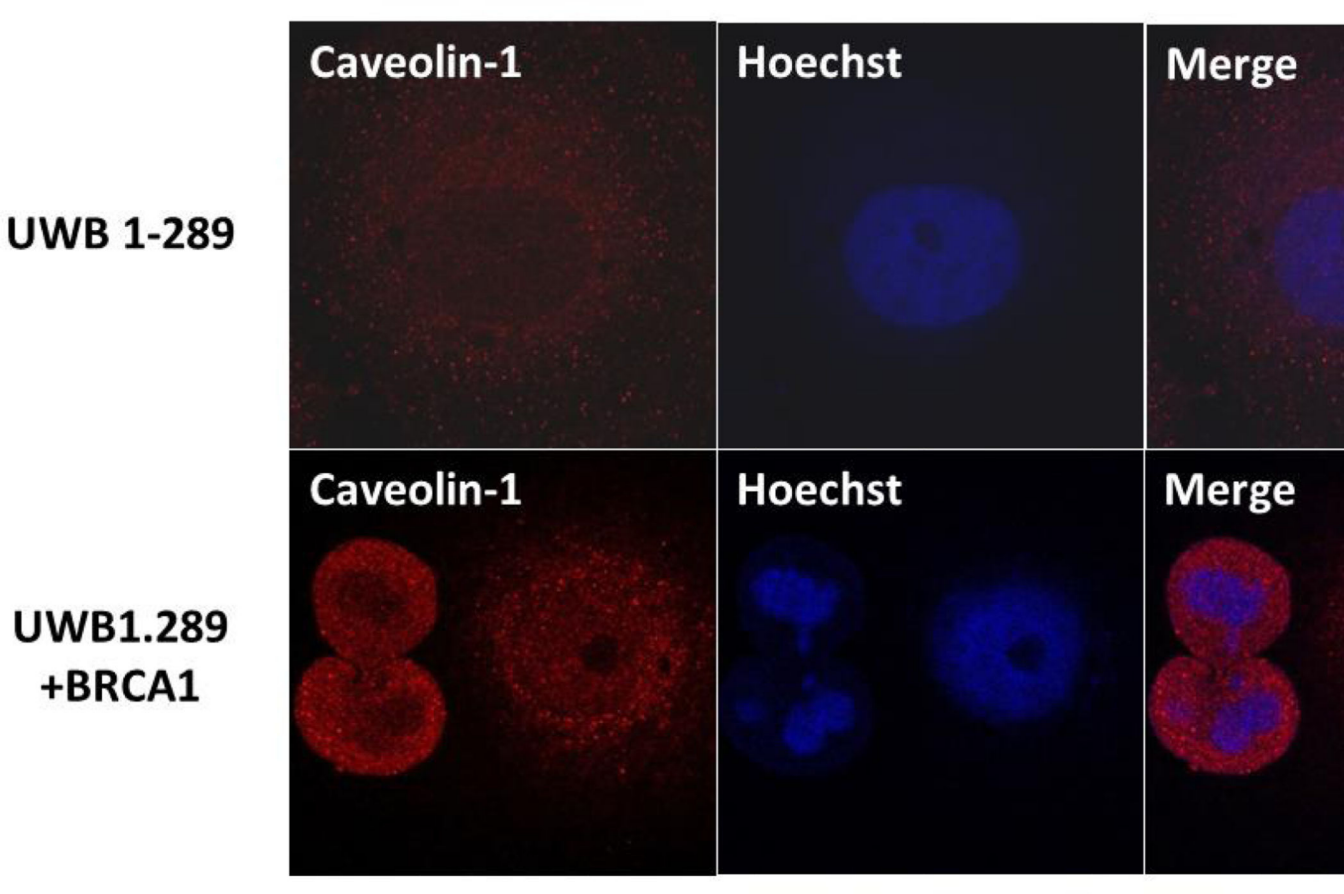
Caveolin-1 is expressed at very low levels in BRCA1 mutant SEOC cells UWB1.289 cells and BRCA1 induces caveolin-1 expression in UWB1.289 cells as detected by immunofluorescence analysis. UWB1.289 and UWB1.289+BRCA1 cells were seeded into six-well plates. The nuclei were visualized using DNA staining dye Hoechst. Cells were fixed in ice cold methanol and probed with caveolin-1 antibody (Santa Cruz, Caveolin-1 1/250) followed by Alexa Fluor 568 labeled secondary antibody (Invitrogen, 1/200) as described previously [34]. The nuclei were visualized by Hoechst staining. The images were taken using LSM 700 Confocal Microscope 63× oil lense, Carl Zeiss).

Caveolin-1 is a major structural component of caveolae and participates in many physiological functions [49–51]. Immunohistochemistry revealed expression of caveolin-1 in normal and benign ovarian epithelial cells, but loss of expression in serous ovarian carcinomas [46]. We therefore, initially examined the expression of BRCA1 and caveolin-1 in a BRCA1-mutant human ovarian cancer cell line, UWB1.289. UWB1.289 is a BRCA1-null ovarian cancer cell line obtained from a papillary serous tumor [48]. This cell line carries a germline BRCA1 mutation within exon 11 and has a deletion of the wild-type allele. Immunoflourescence analysis using BRCA1 antibody showed very low levels of expression of BRCA1 in UWB1.289 cells compared to UWB1.289 BRCA1 cells (Figure1). Similarly immunoflourescence analysis using caveolin-1 antibody revealed low level expression of caveolin-1 in UWB1.289 cells (Figure 2). These results suggest that loss of BRCA1 in UWB1.289 can down regulate caveolin-1 expression similar to what was observed previously in serous ovarian carcinomas [48].

### Wild Type BRCA1 Protein Induces Caveolin-1 Expression in BRCA1 Mutant SEOC Cells

Since we observed low levels of expression of caveolin-1 in BRCA1 mutant UWB1.289 cells and if this is due to loss of BRCA1 then introducing wild type BRCA1 into these cells should induce expression of caveolin-1. We studied the expression of caveolin-1 in UWB1.289 and UWB1.289 BRCA1 cells by immunoflourescence analysis using caveolin-1 antibodies. We observed high levels of expression of caveolin-1 in UWB1.289 BRCA1 cells compared to parental UWB1.289 cells (Figure 2). We also observed a more concentrated membrane staining in UWB1.289 BRCA1 cells (Figure 2). These results are consistent with what was observed previously by Wang et al. [47] in BRCA1+/+ MEF cells.

### BRCA1a but not Ubc9 Binding BRCA1a Mutants are Able to Induce Caveolin-1 Expression in BRCA1 Mutant SEOC Cells

**Figure 3:**
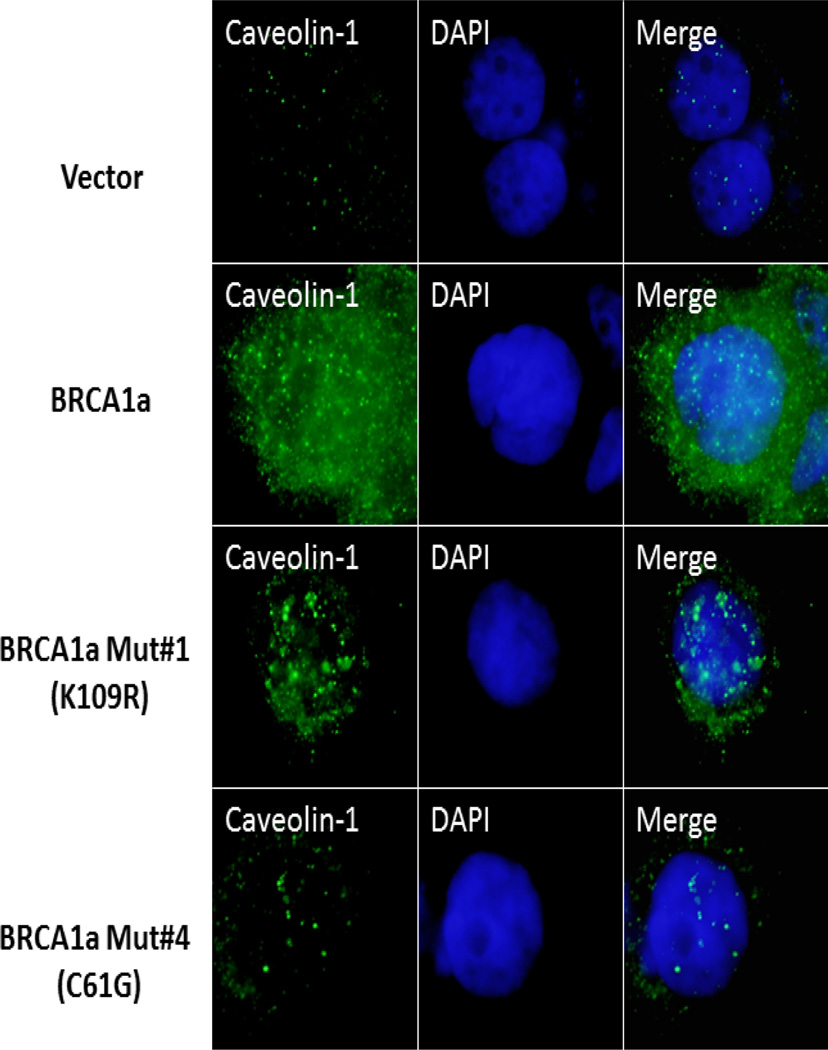
Wild type BRCA1a but not the Ubc9 binding mutants induce caveolin-1 expression in BRCA1 mutant SEOC cells, UWB1.289 by immunofluorescence analysis. UWB1.289 cells were seeded into six-well plates and transfected with pcDNA3 or pcDNA3 BRCA1a or pcDNA3 BRCA1a Mut#1 or pcDNA3 BRCA1a Mut#4 using X-tremeGENE 9 DNA transfection reagent (Roche). The nuclei were visualized with DNA staining dye DAPI 24 hours after transfection. Cells were fixed in ice cold methanol and probed with caveolin-1 antibody (Santa Cruz, caveolin-1 1/250) followed by Alexa Fluor 488 labeled secondary antibody (Invitrogen, 1/200) staining as described [34]. The nuclei were visualized by 4, 6-Diamidino-2-Phenylindole (DAPI) staining. The images were taken using fluorescent microscope (100×, oil Olympus).

**Figure 4:**
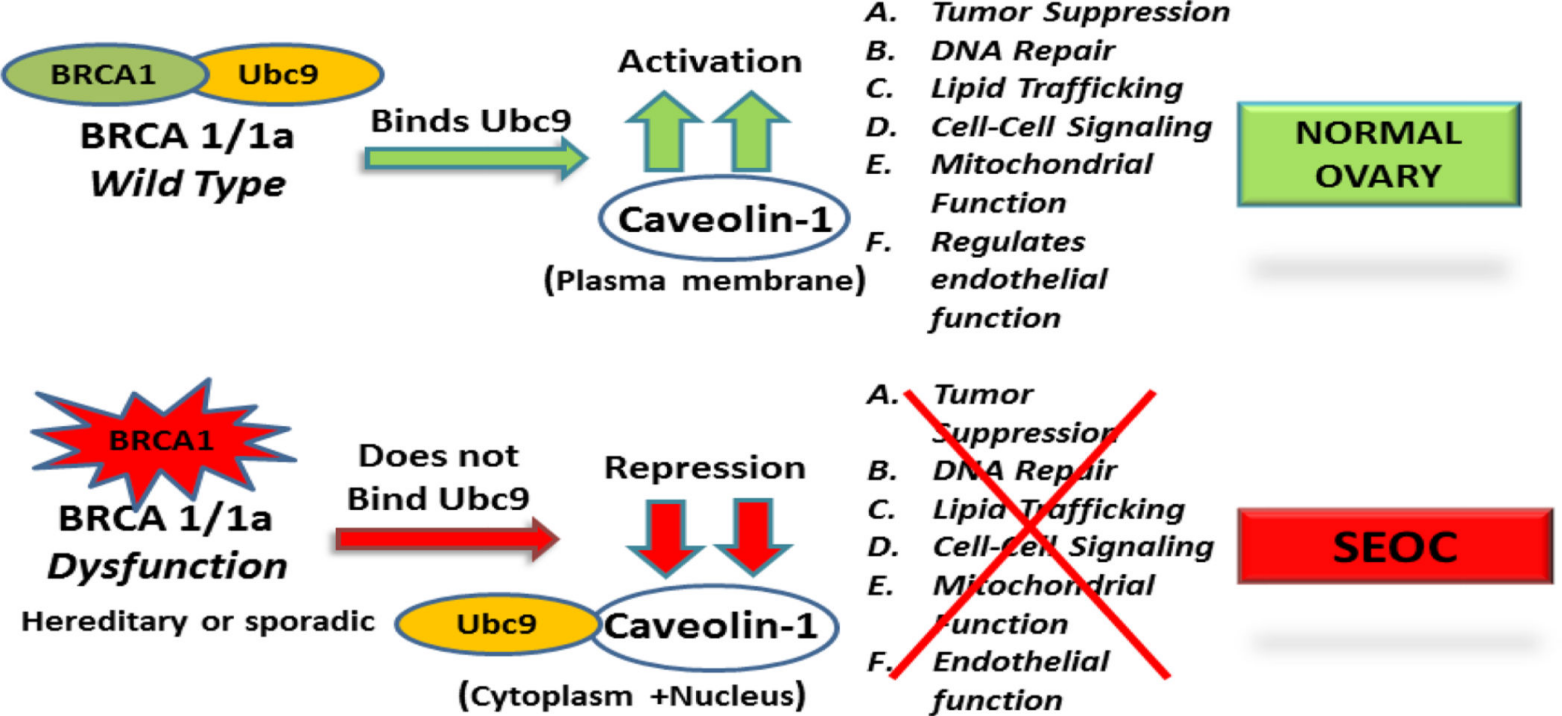
Working hypothetical model showing how BRCA1/1a binding to Ubc9 regulates caveolin-1 expression and function in normal ovaries. In SEOC with BRCA1 dysfunction, Ubc9 is unleashed which inhibits caveolin-1 expression causing loss of DNA repair, lipid trafficking, cellular signaling, endothelial and mitochondrial function resulting in SEOC.

BRCA1 and BRCA1a proteins inhibit the growth of human breast and ovarian cancer cells [16,52–56]. By subjecting BRCA1a, their corresponding Mut#1 K109R, and cancer-predisposing Mut#4 C61G to colony suppression assays using ovarian cancer cells, we were able to show the requirement of Ubc9 binding on the growth suppressor function of BRCA1a proteins in ovarian cancer cells [16]. We wanted to test whether Ubc9 binding by BRCA1 proteins has anything to do with inducing caveolin-1 expression in UWB1.289 cells. We transfected UWB1.289 cells with BRCA1a, BRCA1a Mut#1 and BRCA1a MUT#4 and studied the expression of caveolin-1 by immunoflourescence analysis using caveolin-1 antibodies. We observed high expression of caveolin-1 in BRCA1a transfected UWB1.289 cells compared to BRCA1a Mut#1 and BRCA1a Mut#4 transfected cells (Figure 3). Furthermore we also observed redistribution of caveolin-1 to the plasma membrane unlike the two BRCA1a mutants. These results are consistent with the notion that a direct association of BRCA1a proteins with Ubc9 is critical for inducing the expression of caveolin-1 in SEOC cells. These results also suggest that a direct interaction of BRCA1 with Ubc9 may be needed for growth/tumor suppression by BRCA1 /1a proteins and lack of binding results in deregulated Ubc9 levels causing SEOC (Figure 4).

## Discussions

Women who have a mutation in the BRCA1 gene have an increased risk of developing EOC. High grade serous carcinoma (HGSC) is the most common and lethal histotype associated with germ line BRCA1 mutation. Recent evidence suggests that the fallopian tube could be the most likely tissue of origin of HGSC [7]. Investigating the functional significance of loss of BRCA1 in women with ovarian cancer is critical to understanding how BRCA1 dysfunction results in ovarian cancer. Caveolin-1, a tumor suppressor is the major structural protein of caveolae and plays a critical role in the regulation of various physiological [47,49–51] and pathological processes such as cardiovascular diseases, cancers and neurological disorders. Immunohistochemistry demonstrated expression of caveolin-1 in normal and benign ovarian epithelial cells, but loss of expression of caveolin-1 was seen in SEOC [46]. BRCA1 up regulates caveolin-1 mRNA levels via tethering caveolin-1 promoter in MEF’s cells [47].

For the first time, we are demonstrating low levels of expression of caveolin-1 in the UWB1.289 cells and high levels of caveolin-1 in UWB1.289 BRCA1 cells using immunofluorescence analysis. These results are in agreement with work done by others in MEF cells [47]. We also observed up regulation of caveolin-1 in UWB1.289 cells that have been transfected with BRCA1a and very low levels in BRCA1a K109R and disease associated C61G mutant proteins. Additionally BRCA1/BRCA1a expression led to the distribution of caveolin-1 more to the plasma membrane unlike the Ubc9 binding BRCA1a Mut#1 and BRCA1a Mut#4. As demonstrated by us earlier, the Ubc9 binding mutants fail to bind Ubc9, lack E3 Ubiquitin ligase activity, fail to suppress the growth of ovarian cancer cells and are mislocalized in the cytoplasm of ovarian cancer cells [34]. These results demonstrate for the first time that BRCA1/BRCA1a proteins need to tether Ubc9 in order to induce expression of caveolin-1 in SEOC cells. BRCA1 dysfunction as seen in sporadic SEOC could unleash Ubc9 resulting in down regulation of caveolin-1 expression causing loss of multiple physiological functions of caveolin-1 (like tumor suppression, DNA repair, lipid trafficking, cellular signaling, endothelial and mitochondrial function) resulting in tilting the balance towards ovarian cancer (Figure 4). Since caveolin-1 was found to translocate to the plasma membrane in the presence of BRCA1/BRCA1a proteins, we can speculate that this may provide an important mechanism for regulating the tumor suppression function in sporadic ovarian cancers where somatic mutations in BRCA1 are rarely found. Future efforts will be directed towards understanding whether BRCA1/BRCA1a proteins use Ubc9 binding as a “switch” to control caveolin-1 expression enabling rapid and tight regulation of ovarian cell growth. This work could help in identifying biomarkers to detect ovarian cancer earlier thus reducing the mortality associated with high grade SEOC.
